# Oncology nursing education and practice: looking back, looking forward and Rwanda’s perspective

**DOI:** 10.3332/ecancer.2020.1079

**Published:** 2020-07-24

**Authors:** Marie Goretti Uwayezu, Ruth Sego, Bellancille Nikuze, Margaret Fitch

**Affiliations:** 1 School of Nursing and Midwifery University of Rwanda, College of Medicine and Health Sciences, University of Rwanda, Kigali, Rwanda; 2 Rory Meyer’s College of Nursing, New York University, New York, USA

**Keywords:** oncology nursing practice, oncology nursing education

## Abstract

**Background:**

Oncology care is a highly specialised division of nursing which requires a higher level of training and education following basic preparation. Rwanda, a developing country, initiated education of oncology nurse specialists in 2015. This paper highlights the experience of establishing the programme.

**Methods:**

Selected literature and expert oncology nurses were consulted to provide direction for the development of this paper. The websites of oncology nursing organisations and the curriculum used by the University of Rwanda for preparing oncology nurses were also reviewed.

**Results:**

In 2015, Rwanda initiated the training of oncology nurse specialists (master’s level). The programme has had two successful cohorts graduating. This programme is implemented in a module system with 14 modules. The modules emphasised on screening and diagnosis of different cancers and their treatment, management of treatment related side effects, palliative care, end-of-life care and rehabilitation. A part this formal education, Rwanda, through Partners in Health and the Rwanda Biomedical Center, is also offering in-service training of nurses on cancer treatment, preventive measures and early identification such as Clinical Breast Examination and screening of cervical cancer.

**Conclusion:**

Oncology nurses can play a key role in the care of cancer patients and prevention activities when they have the appropriate education. Rwanda’s experience in establishing a master’s programme in oncology nursing could be of assistance to others who wish to develop a similar programme.

## Introduction

The number of individuals diagnosed with cancer is projected to increase rapidly over the next decade reaching 22 million new cases and 13 million deaths worldwide by 2030 [1]. Sixty percent of the new cases and 70% of the deaths will occur in low- or middle-income countries, creating considerable challenge for the respective healthcare systems [2]. Currently, cancer is the third leading cause of mortality in Africa following communicable and cardiovascular diseases, but 22% of African countries lack access to cancer care [1] and leaders are calling for concerted attention to cancer control, including preparation of healthcare professionals who will be able to deal with the emerging crisis.[3, 4]

Oncology nursing care is a highly specialised subset of practice that requires a higher level of training and education following basic preparation [5]. When nurses have the opportunity to learn about cancer care, their specialised knowledge and skill can influence the quality of care for patients and families. Their participation in cancer screening and cancer care programmes can assist in reducing the burden associated with cancer [6]. In the developed countries, the preparation of oncology nurses as a specialty was initiated in the early 1970s. In Rwanda, a developing country, education of oncology nurse specialists began in 2015 through a collaboration between the USA/Human Resources for Health (HRH) Faculty, University of Rwanda/School of Nursing and Midwifery and Rwandan Ministry of Health.

## Purpose

This paper highlights the development of oncology nursing education in Rwanda which may be helpful to other developing countries in their pursuit of cancer nursing.

## Methods

The preparation for this paper included reading the literature found through a search of PubMed and Scopus databases using the MESH (Medical subject Headings) terms ‘oncology nursing practice and education’. Websites of oncology nursing organisations and the curriculum used by the University of Rwanda for training of oncology nurses were also consulted. The paper highlights the historical background of oncology nursing as a specialty on which our experience was based followed by a description of our approach in establishing oncology nursing education in Rwanda.

## Historical background of oncology nursing

According to the European Oncology Nursing Society (EONS), a cancer nurse or oncology nurse is *a qualified nurse who has the authority and full responsibility to provide essential nursing care to people affected by cancer and their families based upon their evidence-based, specialised, ethical and personal knowledge and skill* [7]*.*

Globally, the education and practice of oncology nursing as a speciality started after the development of medical oncology as a speciality in medicine [5]. Before oncology nursing developed as a specialty practice, cancer patients were cared for by general nurses providing bedside comfort care with few technological advances. During the 1970s and early 1980s, as chemotherapy was introduced, the treatment of cancer patients was seen as needing additional knowledge and skill beyond basic practice. There had always been surgery for cancer patients and radiation had been introduced by that time. However, with the introduction of chemotherapy and recognition of the hazards, it posed for patient and staff, more focus emerged on cancer care as a specialty and the delivery of chemotherapy in special centres. [8]

Over the next decade, speciality practices in medical oncology, radiation oncology and surgical oncology developed and grew. In particular, the advent of chemotherapy and the involvement of nurses in its administration and management of side effects stimulated the growth of oncology nursing as a specialty to a great extent [9]. In addition, the introduction of treatments that could be given on an outpatient basis, and the new approaches that were then required for the management of side effects and education of patients pushed the development of nursing in oncology forward [8].

Since then, oncology nursing has evolved significantly. Professional organisations for oncology nursing have emerged, and with them the development of standards and guidelines for practice that outlined responsibilities and expectations for the speciality roles of nurses in cancer care also have emerged [10]. The roles and responsibilities of oncology nurses have transformed from general basic nursing care to advanced practice and oncology nurses are responsible for everything from performing invasive procedures to diagnostic interpretation and screening for cancer prevention [11].

## Role of oncology nurses

Effective cancer management is achieved when there is a multidisciplinary team and well-skilled nurses play a central role in a functioning team. The goal of reducing cancer incidence, increasing survival rate, providing better palliative care and improving the quality of the patient’s life cannot be achieved without nurses’ efforts. Oncology nurses apply early detection programmes, provide treatments and anticipate complications in addition to demonstrating leadership skills and networking in the clinical area [5]. They carry out these cancer control activities in different clinical settings, such as hospital wards, outpatients, primary healthcare, homes and in palliative care and end-of-life (EOL) care units [12]. The nurse has a key role during cancer treatment decision-making process [13]. Oncology nurses not only experience heavy workloads, but also deliver more complex and advanced care to more seriously ill patients [10]. Several studies confirmed that nurses spend more time with patients than other healthcare professionals [11] and oncology patients spend a significant period of time in oncology hospitals. Nurses are responsible for all types of care including EOL care [14].

## Education of oncology nurses

Oncology nursing is clearly conceptualised as a specialised nursing practice that requires a relatively high level of education [5]. In high-income countries (HICs), well prepared oncology nurses have demonstrated a wide-ranging impact across the spectrum of cancer care [5, 6, 10]. To assist in the preparation of competent oncology nurse specialists, oncology nursing competencies and curricula have been developed by several organisations, including the European Oncology Nursing Society (EONS), the Oncology Nursing Society (ONS), the Association of Paediatric Haematology/Oncology Nurses, the Canadian Association of Nurses in Oncology (CANO/ACIO) and the World Health Organization (WHO). Although these competencies and curricula reflect cancer nursing practice within high-income countries, they can be used as a guiding framework for developing competencies and curricula tailored to low- and middle-income countries (LMICs) [15].

## Cancer nursing development in Africa

In Africa, oncology nursing education and practice as a speciality is not yet well developed. The majority of nurses learn how to care for individuals with cancer through in-service training in their healthcare institutions. Several African countries have begun to develop country-specific cancer curriculums but are at different stages [14]. Kenya offers oncology nursing programmes at masters and diploma levels. The countries, such as Tanzania and Zimbabwe, have experimented with initiatives and gained an education opportunity for oncology nursing through Scholars of Europe and North America. Francophone countries in North Africa, such as Algeria and Morocco, adapted this same model with support from the French League Against Cancer and other European cancer agencies. Few research studies on cancer nursing are conducted across the continent [16]. Nurses provide evidence based care by relying deeply on studies conducted in the developed countries [5].

In the developing countries, including Rwanda, oncology nursing education and practice as a speciality is relatively new and more efforts are needed at national and international levels to foster its growth. As these countries design cancer control plans and add cancer treatment resources to their healthcare systems, oncology nurses will be required to enhance the provision of cancer care [17].

As with all LMICs, African countries need workforce capacity–building efforts to educate nurses in cancer care, expand the scope of nursing practice and increase task-sharing care [14]. Nurses trained as specialists in oncology could address public-health cancer risks, such as smoking and obesity, as well as cancer-causing environmental and occupational hazards in their local areas [18].

As nurses form the main part of the healthcare workforce, they are a natural choice to lead cancer control activities in various settings as they are often the first healthcare provider of contact in many countries, including African communities [19]. Oncology nurse researchers in LMICs could collaborate with epidemiologists, medical anthropologists, environmental health scientists, public health professionals and health economists to gather the much-needed data to measure the efficacy of cancer prevention activities, cancer incidence and prevalence and outcomes of people treated for cancer. Oncology nurses with master’s and doctoral education contribute through advanced practice, education, and scientist roles [14]. In Rwanda, the positive impact of involving nurses after they have in-service training in cancer control has been evaluated. For instance, a school-based, opt-out HPV vaccination programme in Rwanda utilising nurses reduced the two-decade delay in vaccine introduction between HICs and LMICs to 5 years [20].

## Oncology nursing education and practice in Rwanda

### Historical background of nursing education in Rwanda

In Rwanda, nursing and midwifery education started with missionaries in the 1940s delivering general nursing education and later with the incorporation of nursing education in public and private schools. In 1996, the Kigali Health Institute was established with advanced nursing and midwifery education. The institute awarded an advanced diploma (A1) and later introduced bachelor’s and master’s degrees in nursing [21].

In 2006, Rwanda was identified among 57 countries in the world with a significant shortage of healthcare personnel [22]. After this, Rwandan Vision 2020 set a target of raising the nursing personnel to 20 nurses per 100,000 populations [23]. Increasing the number of nurses and midwives was supported by the Ministry of Health in 2007 by starting five Schools of Nursing and Midwifery. Also, in 2013, all higher learning institutions including the five nursing/midwifery schools, fused in one University of Rwanda under the Ministry of Education [21].

In 2015, the Rwandan Ministry of Health worked with the University of Rwanda and, in collaboration with the USA through HRH Faculty, developed eight tracks within the Master of Science in Nursing curriculum, including oncology nursing.

### Rationale of oncology nursing education in Rwanda

A higher learning institution initiates a new educational programme based on the needs of the population. Rwanda, through the University of Rwanda and the Ministry of Health, considered cancer as a public health issue based on data showing that cancer is a leading cause of morbidity and mortality in Africa, the lack of early detection and access to treatment, and cancers related to infectious agents, such as cervix, liver, Kaposi sarcoma, urinary bladder being among the dominant types in sub-Saharan Africa [24]. In addition, cervical cancer accounted for 21% of the total newly diagnosed cancers in females and liver cancer for 11% of the total cancer cases in males at the time. Cancers such as colorectal, lung, prostate and breast cancer were also becoming more common in low-income countries, including Rwanda, due to the adoption of unhealthy lifestyles associated with economic development, such as smoking, physical inactivity and consumption of calorie-dense food [1].

Oncology nurses can play a key role in many cancer control activities, including cancer prevention, screening, early detection, palliative and EOL care. However, the knowledge, skills and competencies necessary to enact these roles require a correspondingly higher level of training and education. Rwanda has recognised the need for these competent oncology nurse specialists in prevention activities and the care of cancer patients. The result of this recognition was the creation of the oncology track within the Master of Science in Nursing programme.

### Oncology nursing programme experience in Rwanda

In 2015, the University of Rwanda initiated a master’s programme in Nursing with eight specialities, including oncology nursing. This is a two-year, four semesters, postgraduate programme in nursing. This programme has 14 modules which cover cancer screening and diagnosis and treatment of different adult and paediatric cancers, management of treatment-related side effects, palliative and EOL care and rehabilitation. Upon completion, graduates are awarded a Master of Science in Nursing (MScN) degree with a specialisation in oncology nursing.

Our curriculum was developed by drawing on published standards and guidelines for oncology nursing (e.g. ONS, EONS and CANO) and consultation with international experts in the field. Very little cancer related content is provided in undergraduate nursing education in Rwanda, and thus providing content related to the disease and its various treatments, including pharmacology, were necessary. Courses in research methods and advanced nursing practice content were also incorporated. Each student was required to design a research proposal, conduct the study, and publish the result.

In addition to utilising clinical labs, students were required to fulfil clinical placement expectations. In total, 500 hours were spent in clinical settings involving among others cancer awareness and prevention, diagnosis and treatment, chemotherapy administration, surgery, palliative and EOL care.

The first cohort successfully graduated in August 2017, and the second graduated in November 2019. The third cohort started at the end of September 2019. The graduate oncology nurses are now absorbed into the healthcare system to improve the quality of cancer care. Some of the graduates are currently allocated in different clinical settings that care for cancer patients and others are in different academic institutions, including the University of Rwanda.

In addition, community outreach events are conducted with the students to create awareness about cancer and its prevention in collaboration with various government and non-governmental stakeholders. Students are engaged actively to participate in such events and carry out cancer screening, especially of the breast.

We have also reviewed the oncology nursing curriculum as of May 2019. It is important to ensure the content is current and aligns with international standards of oncology nursing.

### Continuous professional development for nurses and midwives in practice

In-service training or continuing education of Rwandan nurses and midwives in practice settings about cancer preventive measures and early identification (e.g. Clinical Breast Examination) were started in 2015 at the Butaro Cancer Center of Excellence and its catchment district [25]. The evaluation of the outcomes of this brief training showed that the knowledge about breast cancer and skills in evaluating and managing breast concerns were significantly improved [26]. In addition, since 2013, nurses and midwives from health centres and district hospitals have been trained on screening for cervical cancer using visual inspection with acetic acid through a 10-day curriculum: 5 days for theory and 5 days for practice [27].

### Opportunities

The Master of Science in Nursing Programme is implemented by experienced USA/HRH training specialists from around the globe together with Rwandan faculty. Moreover, the oncology nursing track also has a highly qualified and experienced honorary lecturer. Oncology nurse students perform their clinical practice at Butaro Cancer Center of Excellent (BCCOE), an institution offering dedicated excellent cancer care in Rwanda with trained oncology care professionals. BCCOE offers highly specialised cancer care to the Rwandan population and East Africa community at large (e.g. patients from Burundi, Democratic Republic of Congo and Uganda). Among the services provided by the facility are health promotion and cancer prevention, cancer diagnosis, treatment and palliative care. Oncology nursing students have the opportunity to rotate in various departments, work with the experts and develop their practical skills.

Collaboration between the University of Rwanda and other non-governmental organisations, such as Partner’s in Health (Inshuti mu Buzima) and Dana Faber Cancer Center, is another opportunity that facilitates the learning process of oncology nurses in Rwanda, especially in clinical teaching. This collaboration has been of great support to the students and they have benefited from clinical mentoring by Dana Faber expert nurses in oncology﻿.

### Challenges and future plans

Like any new academic programme, we have had some challenges. One is the financial constraints faced by the students. Some students are self-sponsored and also continue to serve in their respective workplaces, balancing the demands of their work and study lives. Another challenge is a lack of scholarship opportunities for PhD studies for faculty. As a result, financial capacity of the faculty to conduct clinical research is still limited. Another challenge is a lack of scholarship opportunities for internships or placements outside the country in order to obtain more expertise in skills not found in our settings (e.g., bone marrow and stem cell transplantation).

The future plan is to continue to prepare oncology nurses at national and international levels and provide continuous professional development for healthcare providers in healthcare settings. We expect the educational level of the Rwandan academic staff will be increased, and more cancer-related studies will be conducted, published in reputable journals and presented in scientific conferences. We also hope to initiate exchange programmes between Rwandan oncology nurses and international organisations of oncology nurses.

## Conclusion

The establishment of the master’s programme in Rwanda for oncology nurses was undertaken to prepare specialists in the care of cancer patients. Our experience could be of assistance to other countries who wish to move in a similar direction. Oncology nurses can play a key role in the care of cancer patients and in prevention activities if they have the relevant educational preparation.

## Authors’ contributions and conflicts of interest

All authors contributed to the final version of the paper and there are no conflicts of interest.

## Funding support

This paper was originally prepared for presentation at the AORTIC Annual Conference held in Mozambique in 2019. Funding was received for the primary author to travel to the conference but not for the preparation of the manuscript itself.
